# The somatic piRNA pathway controls germline transposition over generations

**DOI:** 10.1093/nar/gky761

**Published:** 2018-08-24

**Authors:** Bridlin Barckmann, Marianne El-Barouk, Alain Pélisson, Bruno Mugat, Blaise Li, Céline Franckhauser, Anna-Sophie Fiston Lavier, Marie Mirouze, Marie Fablet, Séverine Chambeyron

**Affiliations:** 1 IGH, CNRS, Univ. Montpellier, Montpellier, France; 2 Institut Cochin, Paris, France; 3 Institut Pasteur, Bioinformatics and Biostatistics Hub, C3BI, USR 3756, IP CNRS, Paris France; 4 ISEM, CNRS, Univ Montpellier, Montpellier, France; 5 LGPD, CNRS, Univ Perpignan Via Domitia, Perpignan, France; 6 Université de Lyon; Université Lyon 1; CNRS; UMR 5558, Laboratoire de Biométrie et Biologie Evolutive. 43 Boulevard du 11 novembre 1918, 69622 Villeurbanne Cedex, France

## Abstract

Transposable elements (TEs) are parasitic DNA sequences that threaten genome integrity by replicative transposition in host gonads. The Piwi-interacting RNAs (piRNAs) pathway is assumed to maintain *Drosophila* genome homeostasis by downregulating transcriptional and post-transcriptional TE expression in the ovary. However, the bursts of transposition that are expected to follow transposome derepression after piRNA pathway impairment have not yet been reported. Here, we show, at a genome-wide level, that piRNA loss in the ovarian somatic cells boosts several families of the endogenous retroviral subclass of TEs, at various steps of their replication cycle, from somatic transcription to germinal genome invasion. For some of these TEs, the derepression caused by the loss of piRNAs is backed up by another small RNA pathway (siRNAs) operating in somatic tissues at the post transcriptional level. Derepressed transposition during 70 successive generations of piRNA loss exponentially increases the genomic copy number by up to 10-fold.

## INTRODUCTION

Large fractions of the genome of eukaryotes are made of families of parasitic DNA sequences that are able to replicate by transposing into new locations of the host genome (1). These Transposable Elements (TEs) are broadly classified in two classes, retrotransposons and DNA transposons, depending on which nucleic acid intermediate (RNA and DNA, respectively) is used during the transposition process. Moreover, the long-terminal-repeat (LTR) and non-LTR retrotransposon subclasses differ by their genomic structure and retrotransposition mechanism (2,3). We refer to the *Drosophila* LTR retrotransposons as endogenous retroviruses (ERVs) in analogy to the retroviruses that managed to colonize the mammalian germinal genomes.

Mechanisms that maintain TE transposition at a low rate compatible with the survival of the host have been selected during evolution. The repression of TE activity in the gonads of animals is especially important as germline insertions are transmitted to the progeny and thus increase the genomic copy number. However, several exceptions have been reported of *Drosophila melanogaster* females exhibiting unusually high levels of germinal transposition of a given TE family. This happens, for instance, in females from hybrid dysgenic crosses that suffer from derepression of either a DNA transposon (4,5) or a non-LTR retrotransposon (6). Observations of increased transposition rates also include the case of two strains where either of two ERV families is derepressed in the somatic support cells of the ovary: in both cases, the somatic expression is assumed to lead to the production of virus-like particles that are able to infect the female germline and cause new proviral integrations in the progeny (7–9).

These genetic models have allowed to uncover a very elegant small RNA-based mechanism of TE repression in *Drosophila* ovaries. Ovaries where a given TE was derepressed were found to specifically lack the subpopulation of 23–30 nucleotide (nt)-long RNAs that are precisely complementary to this TE family (10–14). These small RNAs have been called Piwi interacting RNAs (piRNAs) because they are loaded on proteins of the PIWI subfamily (10,15). Depending on which PIWI protein they are associated with, Piwi or Ago3 and Aub, they can hybridize with either the nascent or the cytoplasmic transcripts of TEs. This results in the transcriptional (TGS) or post transcriptional (PTGS) TE silencing, respectively (14,16,17).

In the *Drosophila* female germline, the impairment of the piRNA pathway by disruption of the piRNA biogenesis machinery results in transcript accumulation for many TE families at the same time (10,18–20). In the somatic tissues TEs are further repressed by another class of small RNA, called siRNAs (21–25). This pathway regulates TEs at the post-transcriptional level. In S2 cells and in *Drosophila* heads, it has been reported that the impairment of the siRNA pathway effectors result in TE transcript accumulation (24). However, the somatic TE transcript accumulation has never been correlated with *de novo* TE insertions. In the germline, only few studies have examined the consequence of TE transcript accumulation on the host genome. Indeed, it has been reported that TE transcript accumulation could be correlated with *de novo* insertion of one TE family, the P element, in the germline (26,27). These studies suggest that a loss of piRNA regulation at TGS and/or PTGS level in the germline might result in an increase of TE copy number in the next generation.

In contrast to other somatic cells, the somatic cells that surround the *Drosophila* female germline, called follicle cells, possess a simplified piRNA-mediated TE repression pathway (28,29). In these cells, Piwi is required for the transcriptional repression of the ERV families and the depletion of Piwi in follicle cells results in an accumulation of ERV transcripts (30,31). Moreover, a population of 21nt long siRNA-like RNAs mapping on the sequence of one TE has been reported upon impairment of the piRNA pathway in follicle cells (31). This suggests that in follicle cells both siRNA and piRNA pathways coexist. However, the role of siRNAs in TE regulation and the interdependence of these two pathways have never been studied. Moreover, at a genome wide scale, the global impact of ERV family activation in follicle cells on germline genome integrity is still elusive.

Here, we reveal that Piwi depletion, in the follicle cells, leads to a dramatic loss of the piRNAs specifically targeting ERV families. This decrease in piRNAs correlates with an accumulation of steady state ERV RNA levels. Up to 20% of the ERV families that were transcriptionally activated were able to infect the germline and new insertions in the progeny were detected for at least two of these ‘infectious’ ERV families. We demonstrate that the intermediates of transposition detected in the somatic cells and in the early embryos do not necessarily correlate with transposition events. Moreover, multiple levels of TE control reduce the impact of ERV somatic derepression on transposition in the germline. We demonstrate that the siRNA pathway in the somatic cells reduces ERV RNA accumulation and - though less efficient—is a backup for the piRNA pathway mediated TE repression. Over successive generations of piRNA loss the copy number of some ERV families increases exponentially.

## MATERIALS AND METHODS

### *Drosophila* stocks

Fly stocks were maintained in standard conditions (20°C) unless indicated otherwise. A list of the fly lines and crosses employed can be found in Supplementary Table S1.

### Egg hatching test

Twenty five freshly hatched females and males from the piwi-sKD line were shifted from 20°C to 25°C in a collection cage. After 4 days during which the food plates were changed every day, eggs were collected from 4–5, 5–6, 6–7, 7–10 or 10–13 days old females. The egg collections were left to develop at 25°C for further 48 h and the percentage of hatched eggs was determined by counting. Three biological replicates were performed.

### RNA extraction and qRT-PCR

Total RNA from fresh ovaries was extracted with TRIzol (Thermo Fisher Scientific) following the manufacturer's instructions. RNA quantity was accessed with a Nanaodrop, RNA was DNase treated and 0.5 μg RNA were reverse transcribed using 1 μl SuperScript III (Invitrogen) with random hexamer primers in 10 μl reaction volume for 1 h at 50°C. Quantitative PCR analyses were performed with the LightCycler480 SYBR Green I Master mix system with 0.2 μl of cDNA and 0.3 μM of primer mix in 10 μl reaction volume on a Roche LightCycler 480 instrument. RNA levels were calculated with the advanced relative quantification method (Roche) using the *Rpl32* housekeeping gene as reference (for primer sequences, see Supplementary Table S2). Data were analyzed with the LightCycler software (Roche). Each experiment was performed with biological triplicates and technical duplicates.

### RNA purification and sequencing

Total RNA from 30 pairs of ovaries was isolated using TRIzol (Thermo Fisher Scientific), DNAse treated and further purified using Dynabeads™ Oligo(dT)_25_ (Thermo Fisher Scientific). Libraries of the purified polyA mRNA were generated with the TruSeq RNA Library Prep Kit (Illumina) and 50nt single read sequencing was carried out by the Donnelly Sequencing Center (Toronto) on an Illumina HiSeq2500. The RNA-seq experiments were performed with two biological replicates.

### DNA extraction and qPCR

Genomic DNA was extracted from 3 to 17 h bleached embryos with the GenElute Kit (Sigma G1N70) following the manufacturer's instructions. One nanogram of total DNA was used for quantitative PCR reaction which was performed with the LightCycler 480 SYBR Green I Master mix on a Roche LichtCycler 480 instrument. Relative TE copy number was calculated with the advanced relative quantification method (Roche) using the *Rpl32* single-copy gene as reference (for primer sequences see Supplementary Table S2). Data were analyzed with the LightCycler software (Roche). Each experiment was performed with biological triplicates and technical duplicates.

### DNA purification for sequencing and PCR

Genomic DNA from at least 50 μl of 3–17 h bleached embryos was extracted with the PureLink Genomic DNA Mini Kit following the manufacturer's instructions (Invitrogen cat. K1820-01). 100nt paired-end read sequencing were performed by BGI (China) on an Illumina HiSeq2500. Total DNA from individual adult flies was isolated using the GenElute Kit (Sigma G1N70). Library was prepared using the NEXTflex Rapid DNA Sequencing Kit and 125 nt paired-end sequencing was performed on an Illumina Hiseq 2500 by Montpellier Genomix (France). For verification, 10 ng of the single fly DNA were used for PCR with GoTaq HotStart Polymerase (Promega) following the manufacturer's instructions (for primer sequences see Supplementary Table S2).

### Oxidation of hydroxyl-groups

Small RNA oxidation was done as described in (32). Briefly, 12 μg of total RNA from piwi-sKD ovaries were incubated in borate buffer (29.6mM, 29.6 mM boric acid pH 8.6, 0.5% SDS) with NaIO_4_ solution (25 mM) for 30 min at RT. Then 3 μl of 100% glycerol was added for 10 min at RT to quench the remaining NaIO_4_. RNA was further purified on Roche mini Quick-Spin Oligo column and ethanol-precipitated in the presence of 20 μg Glycoblue. We have then quantified the population resistant to this treatment as previously described in (33) with the following modifications: 100 ng of total RNA was polyadenylated and reverse transcribed in the same reaction tube with *Escherichia coli* Poly(A) Polymerase (M0276S, NEB), Superscript II (Invitrogen) reverse transcriptase and the 5′-CAGGTCCAGT15VN primer. The reaction was incubated at 37°C for 10 min, then at 42°C for 50 min and finally at 70°C for 15 min. Primer sequences used for RT-qPCR are listed in Supplementary Table S2.

### Small RNA purification and sequencing

Small RNAs from ovaries were isolated on HiTrap Q HP anion exchange columns, as previously described (34). Small RNAs were size selected (18–30nt) on gel, 50nt single-read sequencing on an Illumina HiSeq2000 apparatus was performed by BGI (China) for no-KD and piwi-sKD and by Donnelly sequencing Center (Toronto) for piwi-sKD, dcr2-sKD and piwi-sKD, ctrl-sKD.

### Mobilome-seq and Mobilome-PCR

The sequencing of the eccDNA from *Drosophila* embryos was done as described in (35). Briefly, genomic DNA was extracted from 0–2 h embryos using the DNeasy Blood & Tissue Kit (Qiagen) and further purified with the PCR purification kit (Qiagen). Linear genomic DNA was digested with Plasmid-Safe™ ATP-Dependent DNase (Lucigen) and the remaining eccDNA molecules were then amplified by rolling circle amplification (RCA) using Illustra TempliPhi kit (GE Healthcare) and random primers. Sequencing libraries were prepared with the Nextera XT library kit (Illumina) and 250 nucleotides paired-end sequencing was performed using the MiSeq platform (Illumina) by Plateau de Génotypage CIRAD (France). For PCR confirmation 10 ng of RCA amplified DNA was used (for primer sequences see Supplementary Table S2).

### Immunofluorescence

Ovaries from young females were dissected and fixed in PBS, 4% formaldehyde, 0.1% Triton-X100 for 20 min, washed in PBS with 0.1% Triton-X100. The primary antibody dilutions for immunostaining were 1:200 for rabbit anti-Piwi (Santa Cruz-98264), 1:8000 for guinea pig anti-tj (gift from D. Godt, Toronto) and 1:1000 for rabbit anti-dcr2 (gift from F. Gebauer, Barcelona) antibodies. The anti-rabbit and anti-guinea pig secondary antibodies were 1:400 and 1:200 diluted, respectively. Ovaries were Dapi stained and mounted in Vectashield medium (Vector Laboratories). Fluorescent images were acquired with a Zeiss Apotome microscope. Images were treated in Omero (https://www.openmicroscopy.org/omero/).

### Computational analysis

#### Small RNA-seq

Raw reads were trimmed from their 3′ linkers. Trimmed reads (18–30nts in size) were mapped with Bowtie2 (36) using mismatch-tolerant settings to the *Drosophila melanogaster* genome (release 5; dm3) complemented with canonical transposable elements (TEs) (*Drosophila* consensus TE sequences taken from https://github.com/cbergman/transposons). Reads were annotated based on their mapping coordinates. Small RNAs mapping on piRNA clusters (10), ovary siRNA clusters (22), TEs or 3′UTR of coding genes (ftp://ftp.flybase.net/), and not to rRNAs or miRNAs were defined. Candidate piRNAs were a subset of the above defined reads with a size between 23 and 30 nucleotides. Candidate siRNAs were a subset of the above defined reads with 21nt. Unique mappers were defined as reads for which only one best-score alignment existed in the genome. Candidate piRNAs were mapped again on piRNA cluster sequences and canonical TE sequences. Candidate siRNAs were mapped again on ovarian siRNA cluster sequences and canonical TE sequences. Data were normalized using the unique mapper reads of piRNA cluster 1 (42AB). Our full analysis pipeline is available at https://bitbucket.org/blaiseli/pirna-pipeline.

#### RNA-seq TE transcript analysis

RNA-seq reads were trimmed for quality using UrQt (using parameter –t 20) (Modolo and Lerat, 2015), and aligned against *D. melanogaster* genes (ftp://ftp.flybase.net/genomes/Drosophila_melanogaster/dmel_r6.09_FB2016_01/fasta/dmel-all-gene-r6.09.fasta.gz) using TopHat2 (37). To generate a gene count table alignment, counts were performed on sorted bam files using eXpress (38). In parallel, we computed a TE read count table using the TEcount module of TEtools (39) on a list of TE copies retrieved from the *D. melanogaster* sequenced genome (available upon request). Gene count table and TE count table were then concatenated for normalization purposes and analyzed using DESeq2 (version 1.10.) (40).

#### Mobilome-seq

Mobilome reads were mapped with Bowtie2 version 2.2.4 (using parameters -k 10 -N 1) on the *Drosophila melanogaster* genome (dm3) complemented with canonical TE sequences. Reads having their best-score alignment either in genomic copies of TEs or canonical TEs were annotated as TE reads. TE annotated reads were then mapped again using only the canonical TEs as references, with Bowtie2 and the same parameters as above, to assign each read to a TE family. Read alignments with a mapping score strictly lower than –10 were discarded. If a read had several ex-aequo best-score alignments, the read count associated to each corresponding mapping location was down-weighted accordingly. A normalization by thousands of mitochondrial DNA (mtDNA) reads was applied to account for library depth differences. Scatter plots were done using the pysam (41) and matplotlib (42) Python libraries. Our full analysis pipeline is available at https://bitbucket.org/blaiseli/mobilome.

#### DNA seq and de novo TE insertion analysis

Paired–end sequencing reads were mapped against the *D. melanogaster* genome (dm6) with BWA version 0.7.5a-r405 for the pooled embryo sequencing experiments (one no-KD and one piwi-sKD library), and with BWA (ArXiv 13033997 Q-Bio) version 0.7.10-r789 for the 12 single fly piwi-sKD sequencing experiments. *De novo* TE insertions for both experiments were determined using the T-lex *de novo* pipeline (43,44). T-lex *de novo* uses reference mapped reads to identify partially mapped, so called soft-clipped reads and read pairs for which only one read is mapped successfully, called one-end anchored (OEA) pairs. The non-mapped reads and read parts are clustered according to their genomic position of the mapped read part or read and clusters were aligned using BLAT (45) against a TE library composed of the 600 first and last bases from 123 canonical TE sequences retrieved from flybase (TE library available upon request). Finally, a second clustering step taking into account the BLAT results defines the TE insertion breakpoint on the reference sequence. To determine predicted new insertions unique to each library we did intersections of bedfiles that were produced from the T-lex *de novo* output files. When comparing files two by two bedtools window (option –w20) (46) for multiple comparisons the command bedtools multi-intersect (using an added window of 20 nt up-and downstream of the insertions site) was used.

Library-specific predicted insertions could be explained by either true TE insertions specific to the sequenced genome or by false positives produced by artefactual chimeric TE-genome fragments that occur during library preparation, with current technologies (27). In contrast to real TE-genome chimeric fragments coming from TE-genome borders at insertion points, the artefactual TE-genome chimeras correlate in abundance in the library with the abundance of the TE in the genome, this means that the detection of elements with a high genome load will be more difficult as real chimeric fragments will be diluted by a high number of artefactual chimeras. Artefactual TE-genome chimeras also have break points in the TE that show no preference for TE ends. To reduce the detection of false positives in our analysis we restricted the size of the TE sequences in the library according to the insert size (∼500nt) used during paired-end sequencing. To disfavor false positive prediction of new TE insertions in our analysis we determined the ratio of potential new insertions in the piwi-sKD and the noKD condition for each TE family. With the same genome sequencing coverage, the ratio of predicted new insertions in the piwi-sKD versus the no-kd condition should be above 1 for those TE families, which experienced *de novo* insertions after Piwi depletion, whereas false positive predictions should be as abundant in the no-KD sequencing as in the piwi-sKD sequencing.

In the sequencing experiment for the pooled embryos, we obtained different coverages for piwi-sKD-G1-F2 and no-KD (96.89× and 78.75× respectively). To be able to compare the number of insertions obtained for both data sets we randomly subsampled the number of mapped reads from the Piwi-sKD-G1-F2 data set with samtools view (using option –s) (41) to match the numbers of mapped reads for the no-KD data set 44 times. Each of the subsamples was analyzed with T-lex *de novo* and the results of each analysis used to calculate the mean for all 44 experiments.

### Quantification and statistical analysis

#### Calculation of transposition rate

The transposition rate for ZAM and gtwin was calculated with the number of TEs detected by qPCR or DNA-seq as followed: (# of *de novo* insertions)/(# of pre-existing insertions) × (# of generations).

#### Statistical tests

**Figure 1.**
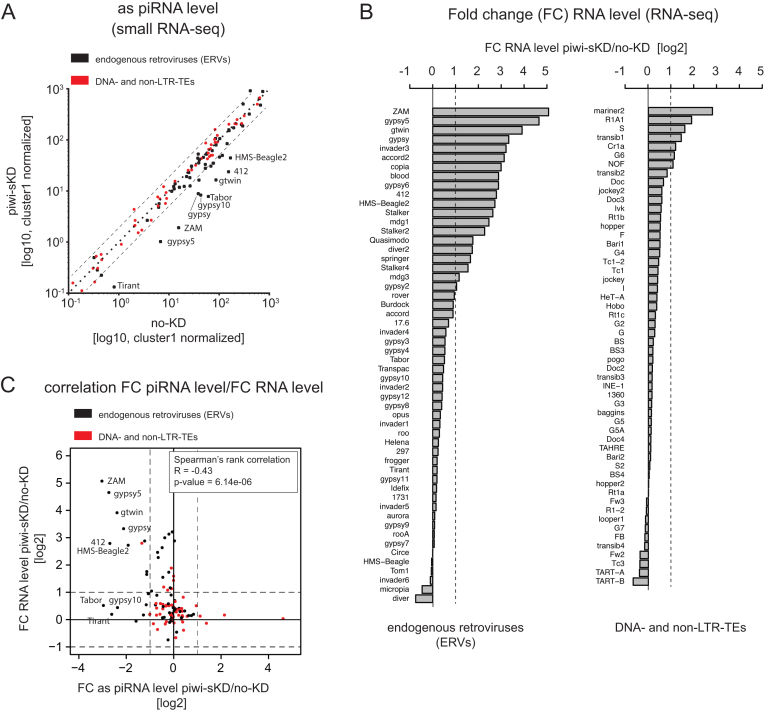
Somatic piwi depletion leads to ERV-specific piRNA loss that correlates with ERV RNA increase. (**A**) Scatter-plot of antisense 23–30 nt RNA-seq reads normalized to the number of cluster 1 unique mappers reads (germline specific piRNA cluster) for annotated Drosophila melanogaster TEs (*n* = 123) in Piwi somatic knockdown (piwi-sKD) versus control (no-KD) (log10 scale; as: antisense). ERVs are depicted as black dots, DNA- and non-LTR-TEs as red dots. (**B**) Bar plot displaying TE RNA level fold changes calculated with normalized read count (two biological replicates) between piwi-sKD versus no-KD ovaries (log2 scale). ERVs are shown in the left panel and DNA- and non-LTR-TEs in the right panel. FC: Fold change. (**C**) Scatter-plot showing the correlation between fold change of normalized TE RNA level (piwi-sKD versus no-KD ovaries) and fold change of normalized antisense TE piRNA reads (piwi-sKD versus no-KD ovaries). ERVs are depicted as black dots, DNA- and non-LTR-TEs as red dots. Correlation was calculated as Spearman rank correlation. FC: Fold change; as: antisense.

**Figure 2.**
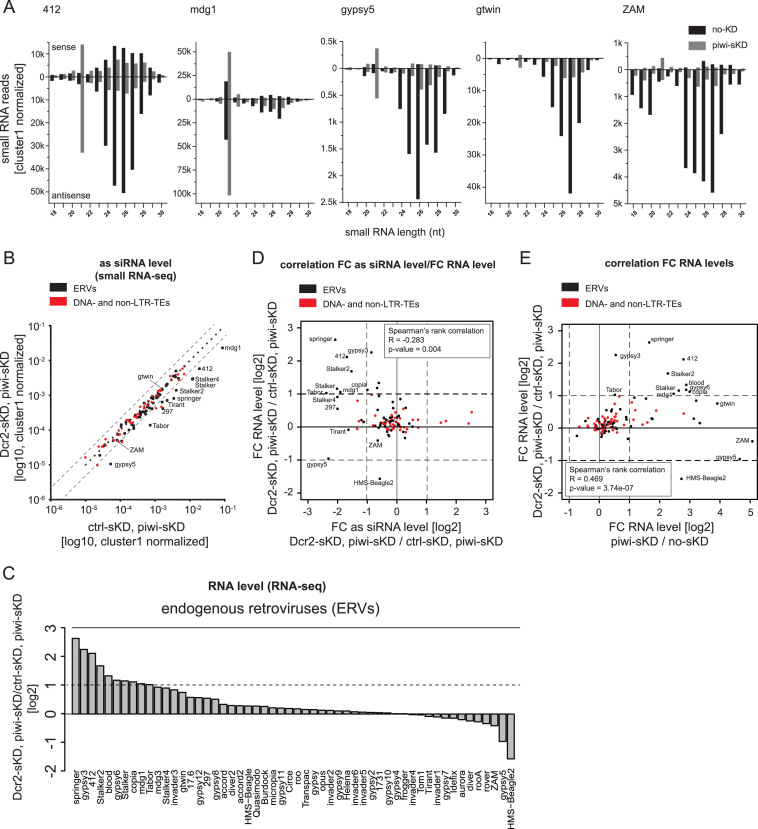
The siRNA pathway participates in somatic ERV repression. (**A**) Size distribution (18–30nt) of sense (displayed above the X axis) and antisense (displayed below the X axis) small RNAs from no-KD ovaries (black bars) and piwi-sKD ovaries (grey bars) mapping to 412, mdg1, gypsy5, gtwin and ZAM. (**B**) Scatter plot comparing the number of antisense 21 nt small RNA-seq reads, normalized to the number of cluster 1 unique mappers (germline specific piRNA cluster), that matched annotated TEs, piRNA clusters or siRNA clusters in Dcr2-sKD; piwi-sKD and ctrl- sKD; piwi-sKD double knock down ovaries (log_10_ scale). The crtl-sKD was a somatic follicle cell RNAi targeting the unrelated white gene. ERVs are depicted as black dots, DNA- and non-LTR-TEs as red dots. (**C**) Bar plot displaying TE RNA level fold changes calculated with normalized read count (two biological replicates) between Dcr2-sKD; piwi-sKD and ctrl-sKD; piwi-sKD double knock down ovaries (log_2_ scale) for the 62 annotated Drosophila ERVs. (**D**) Scatter plot showing the correlation between fold changes of TE RNA level (Dcr2-sKD; piwi-sKD versus ctrl-sKD; piwi-sKD) and fold changes of TE piRNA level (Dcr2-sKD; piwi-sKD versus ctrl-sKD; piwi-sKD) (log_2_ scale). ERVs are depicted as black dots, DNA- and non-LTR-TEs as red dots. Correlation was calculated as Spearman rank correlation. FC: Fold change; as: antisense. (**E**) Scatter plot showing the correlation between fold changes of TE RNA-level in piwi-sKD versus no-KD ovaries and Dcr2-sKD; piwi-sKD versus ctrl-sKD; piwi-sKD ovaries (log2 scale). ERVs are depicted as black dots, DNA- and non-LTR-TEs as red dots. Correlation was calculated as Spearman rank correlation. FC: Fold change.

Data are expressed as mean ± standard deviation (SD). A Spearman rank correlation between the fold changes on RNA level and piRNA level/siRNA level (Figures 1C and 2D) and the fold change in RNA levels (Figure 2E) were determined using R.

A Shapiro-test to determine the normality of the data and a two-tailed t-test were used to determine the significance in differences between qPCR results for the TE-load (Figure 4D and Supplementary Figure S3B) were performed using R and Prism. The *P*-values are *P < 0.05, **P < 0.01 and ***P < 0.001. *P* < 0.05 was considered statistically significant. Probability of observing at least one new ZAM insertion after two generations.

If we assume a germinal transposition rate of 10^−6^ to 10^−4^ for each TE copy at each generation, then (with four initial active ZAM copies) the probability of zero new insertion after one generation is e^−λ^ (with λ = 4 × 10^−6^ to 4 × 10^−4^) (Poisson distribution for *k* = 0). The probability of having zero new insertion after two generations is e^−2λ^ because transposition events are expected to be independent between generations. The probability of observing at least one new insertion in one individual after two generations is 1 – e^−2λ^.

Probability of observing at least 1 out of 12 F2 flies with at least 1 new insertion: we will first calculate the probability of observing 0 fly with at least 1 new insertion. Because the 12 tested flies are independent, that probability is e^−2x12λ^. Probability of observing at least one fly with at least one new insertion: 1 – e^−2x12λ^. Considering λ = 4 × 10^−6^, the result is ∼9.6 × 10^−5^; considering λ = 4 × 10^−4^, it is around 9.6 × 10^−3^.

## RESULTS

### Conditional knockdown of Piwi in ovarian somatic cells leads to ERV derepression while preserving female fertility

The piRNA pathway represses TE activity in gonads. In the follicle cells of *Drosophila* ovaries, the somatic cells that surround the germline cells, this pathway functions with only one Argonaute protein belonging to the PIWI subfamily called Piwi (10,29). The complete depletion of Piwi in the follicle cells during development leads to atrophic ovaries and sterility (31,47). This makes the multigenerational study of the consequences of the loss of the piRNA pathway activity in somatic cells on TE biology and on genome stability impossible with standard genetic approaches.

We therefore developed a Piwi knockdown system that restricts the Piwi depletion in adult follicle cells to a level that preserves female fertility. The fly line carries three components: (i) a GAL4 UAS-activator driven by the follicle cell-specific *traffic jam (tj)-*promoter (tj-GAL4), (ii) a UAS-short-hairpin(sh)-piwi, that induces Piwi RNAi and (iii) a ubiquitously expressed thermo-sensitive GAL4-inhibitor, GAL80^ts^ (see Supplementary Table S1). At 20°C, Gal80^ts^ sequesters GAL4, preventing shpiwi expression. At 25°C, GAL80^ts^ is inactive, allowing GAL4-driven expression of shpiwi in the somatic follicle cells and thus Piwi depletion (Supplementary Figure S1A). Culturing the flies at 25°C during 5 days starting from late pupal to adult stage leads to a strong Piwi depletion while preserving almost normal ovarian morphology (Supplementary Figure S1B) and fertility (Supplementary Figure S1C). We refer to this condition as piwi somatic KnockDown (piwi-sKD).

First we verified that the restricted somatic Piwi knockdown is sufficient to induce changes in the piRNA population. We compared piRNAs from piwi-sKD ovaries with piRNAs from control ovaries by small RNA-seq. As control, we used the same fly line constantly raised at 20°C, and hereafter referred to as ‘no-KD’. Among the 123 TE families studied, a subset of 17 TEs shows a more than 2-fold piRNA loss in the piwi-sKD (Figure 1A). As expected, the majority of these TEs (16/17) belong to the ERV subclass of retrotransposons. The 7 most impacted families (with a more than 5-fold piRNA loss) are ZAM, Tabor, gypsy5, 412, Tirant, gtwin and gypsy10.

We then wondered whether the piwi-sKD has an impact on TE RNA level. To answer this, we used RNA-seq to compare the RNA-levels for 123 *Drosophila* TE families in piwi-sKD and no-KD ovaries. We found 27 TE families with a more than 2-fold increase in TE RNA level in piwi-sKD compared to no-KD. Most of these TE families belong to the ERV subclass of retrotransposons (Figure 1B). We validated the RNA-seq data by studying a subset of TEs by RT-qPCR (Supplementary Figure S1D). We also checked that the piRNA loss induced by our experimental approach is the cause of TE upregulation rather than the temperature shift. To do so, we introduced a non-thermosensitive GAL80 transgene into the piwi-sKD fly line. Flies were housed either at 20°C constantly or shifted for five days from pupal to adult stage at 25°C and the RNA levels of a subset of 13 TEs were compared by RT-qPCR (Supplementary Figure S1E). None of the tested elements showed an increase at the RNA level after the temperature shift, suggesting that, in our experimental conditions, the piRNA loss was indeed the major cause of TE derepression. This connection between piRNA loss and TE RNA upregulation was also supported by a robust anti-correlation (Spearman's rank correlation *R* = −0.43) between the loss of piRNAs and changes in RNA level per TE family (Figure 1C). Consistent with the piRNA loss being the major cause of RNA upregulation, the three elements (ZAM, gypsy5 and gtwin) that showed the strongest RNA accumulation showed also a more than 5-fold loss of piRNAs. However, there are TE families for which a strong piRNA loss does not translate into RNA upregulation (e.g. gypsy10 and Tirant). These piRNAs against seemingly inactive TE families most likely reflect the adaptive immunity response of the piRNA pathway, built up by ancient TE invasions of these families (see Discussion). Altogether our data show that the piRNA pathway activity is required for the transcriptional regulation of many ERVs in the follicle somatic cells.

### The siRNA pathway can attenuate the upregulation of ERV transcripts in somatic-Piwi-depleted ovaries

The piRNA pathway is not the only pathway with capacity to repress TE activity in flies. The siRNA pathway is the major repressor of TEs outside the fly germline (21–25), but it is also active in ovaries (22,48,49) where it can affect the repression of at least some TEs (22). Moreover, one study reported the upregulation of 21 nt-long antisense small RNAs against ZAM in Piwi-depleted follicle cells (31). To look for similar siRNA-like RNAs after piwi-sKD, we analyzed the 21 nt-long RNA populations in the small-RNA-seq data from piwi-sKD and no-KD control ovaries. We could find four ERV families (412, gypsy5, gtwin and mgd1) with a dramatic increase (up to 10-fold) of matching sense and antisense 21 nt-long reads in the ovaries with Piwi-depleted follicle cells (Figure 2A, Supplementary Figure S2A). These four families belong to the subset of those showing piRNA loss and RNA upregulation after piwi-sKD, suggesting an inducible response of the siRNA pathway by the loss of Piwi in the somatic follicle cells. We wondered whether these 21 nt-long RNAs are *bona fide* siRNAs and if they are contributing to TE repression in somatic follicle cells.

We first investigated whether the biogenesis of these 21 nt-long RNAs involves Dicer-2 (Dcr2), the RNAse that processes dsRNA precursors into siRNAs. We performed a Dcr2, Piwi double somatic knockdown by expressing a *Dcr2* short hairpin construct in the piwi-sKD follicle cell background (Dcr2-sKD; piwi-sKD) (see Supplementary Table S1 for genetic crosses). This condition was compared to a ctrl-sKD; piwi-sKD double knockdown. The efficiency of the Dcr2 and Piwi depletions was confirmed by immunofluorescence (Supplementary Figure S2B-C). We analyzed the 21 nt-long RNA populations matching the 123 *Drosophila* TEs by small RNA-seq. We identified 12 ERV families producing 21 nt-long antisense RNAs in the follicle cells in a Dcr2-dependent manner (Figure 2B). These include three (mdg1, 412 and gypsy5) of the four TEs whose siRNAs are increased in the Piwi somatic knockdown.

We then determined if these 21 nt-long RNAs were loaded on Ago2. To do so, we studied the population of small RNAs after oxidation as the small RNAs loaded on Ago2 have been described to be 2′-O-methylated (50). This modification protects the small RNAs from being oxidized. Since this treatment had no effect on the amount of gypsy5 and 412 21-nt long RNAs we concluded that these RNAs are modified and likely to be loaded on Ago2 (Supplementary Figure S2D). As a proxy for Ago2-loaded 21-nt long RNAs, we also checked that, as reported for Ago2-loaded TE-derived siRNAs (32), the gypsy5- and 412-derived 21-nt small RNAs do have a preference for adenosine at their first nucleotide (Supplementary Figure S2E).

To see whether this loss of siRNAs in the Dcr2-sKD; piwi-sKD condition would lead to an increase of TE RNA, compared to the level of TE RNA in the piwi-sKD alone, we performed RNA-seq in Dcr2-sKD; piwi-sKD and ctrl-sKD; piwi-sKD double knockdown ovaries. We found that these somatic ovarian siRNAs contribute notably to TE repression in Piwi-depleted follicle cells: 10 ERV families had a more than 2-fold higher RNA level in the Dcr2-sKD; piwi-sKD than in the ctrl-sKD; piwi-sKD ovaries (Figure 2C and Supplementary Figure S2F). Moreover, 7 of them belong to the subset of 12 TEs producing Dcr2-dependent siRNAs in the follicle cells and the level of the derepression anti-correlates with the extent of the siRNA loss (Figure 2D). For most elements (8 out of 10) the Dcr2-dependent derepression corresponds to an enhancement of the derepression already caused by the loss of Piwi alone (Figure 2E). We confirmed this observation by RT-qPCR for 412 and mdg1 (Supplementary Figure S2G). Conversely, there are also a number of TEs (e.g. gtwin, ZAM and gypsy5) that are only sensitive to the piRNA loss (Figure 2E and Supplementary Figure S2G). In summary, we found that both the piRNA and siRNA pathways jointly control an overlapping subset of TEs in the somatic follicle cells. The repression by the piRNA pathway affects more elements and is generally stronger (up to 32-fold versus up to 6-fold) than the siRNA-mediated repression.

### Impact of somatic Piwi depletion on two steps of the ERV replication cycle: reverse transcription and female germline infection

**Figure 3.**
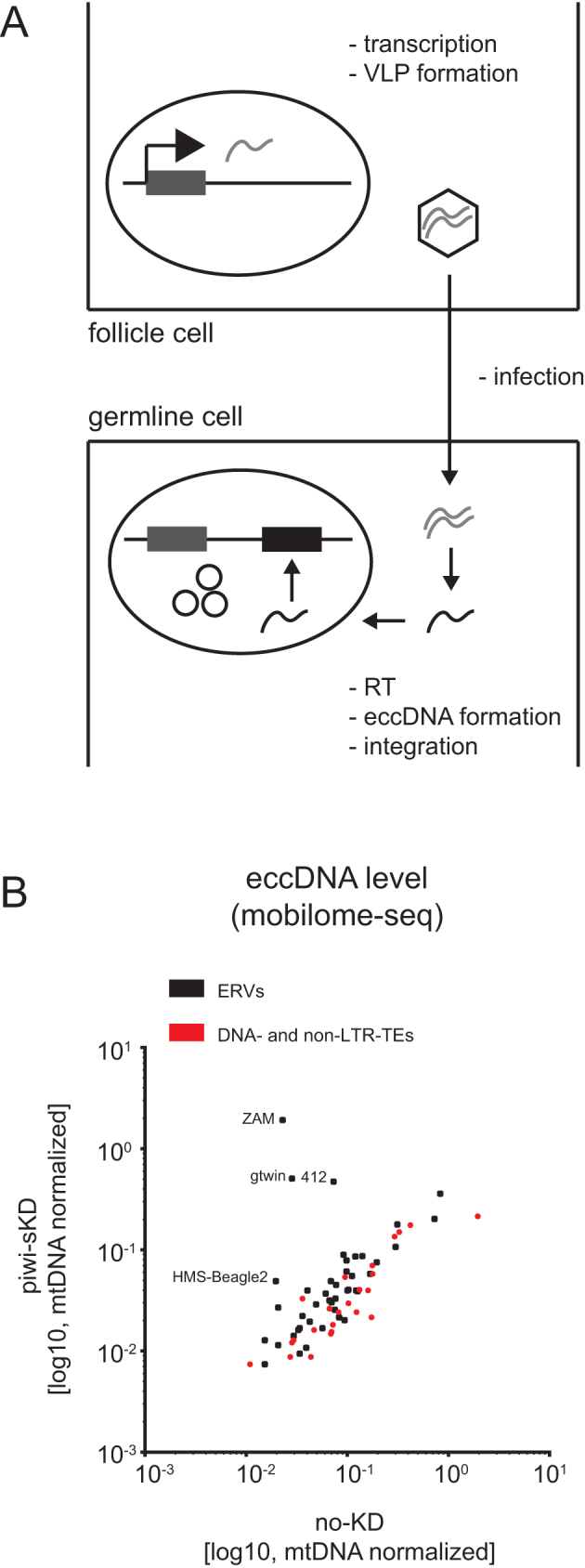
Somatic piwi depletion may lead to ERV virus like particle (VLP) germline infection. (**A**) Schematic representation of ERV life cycle. ERV transcription, translation and VLP formation takes place in the somatic follicle cells. The VLP is then crossing by an unknown mechanism the cell-cell border between follicle cell and germ line cell. RNA is reverse transcribed into cDNA. Viral cDNA enters the germ cell nucleus and integrates. EccDNA is formed as a by-product of integration. (**B**) Scatter plot showing the number of TE mapping mobilome-seq reads, normalized to the number of mitochondrial DNA (mtDNA) mappers, in embryos after piwi-sKD or no-KD (log10 scale). ERVs are depicted as black dots, DNA- and non-LTR-TEs as red dots.

As we observed increased ERV RNA expression after piwi-sKD in follicle cells, we then asked whether this first step of the virus life cycle is followed by production of functional virus like particles (VLPs) and germline infection (Figure 3A). To determine if the ERV families expressed in the somatic cells had infected the germline cells, we performed mobilome-seq in embryos laid by piwi-sKD and no-KD females. Mobilome-seq is a technique to detect extra-chromosomal circular DNA (eccDNA) (35), that can be formed as by-products during retroviral cDNA integration (51–53). Detecting eccDNA suggests in addition that the ERVs have been properly reverse transcribed.

Out of the 20 ERVs that were derepressed at the RNA level, 4 (ZAM, gtwin, 412 and HMS-Beagle2) showed an increased level of eccDNA in the embryos laid by piwi-sKD mothers compared to the level in embryos from no-KD mothers (Figure 3B). We confirmed by qPCR the strong increase of eccDNA species for the ZAM family (Supplementary Figure S2H). The appearance of unintegrated circular viral DNA structures in the embryos demonstrates that these 4 elements have been able to infect the germline and to be reverse transcribed into cDNA, which are crucial steps in the ERV life cycle.

### Somatic Piwi depletion over many generations leads to exponential increase of *de novo* ERV insertions

**Figure 4.**
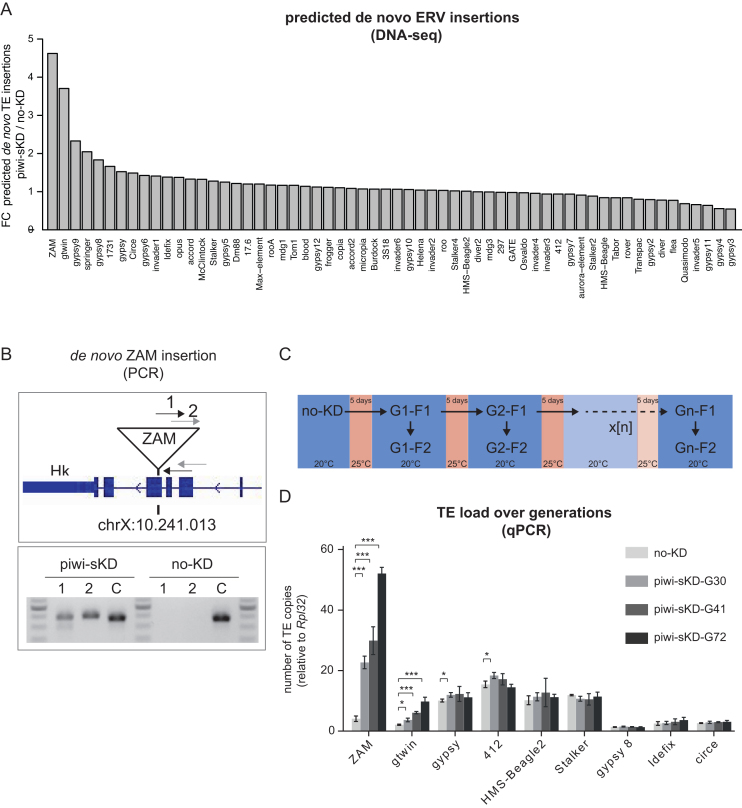
Somatic piwi depletion may lead to de novo ERV insertions in the offspring genome, with a transposition rate stable over generations. (**A**) Bar plot displaying the ratio of *de novo* insertions per ERV family (n = 62) in embryos of the F2 generation after piwi-sKD versus no-KD embryos. (**B**) PCR-validation of a *de novo* ZAM insertion detected by DNA-seq at position chrX:10.241.013. gDNA from flies, of the F2 generation after piwi-sKD, in which the *de novo* ZAM insertion was detected and, as control, their no-KD ancestors was used. The upper panel shows a schematic representation of the genomic location of the de novo ZAM insertion with the two primer pairs spanning the break point between the 5′ end of the insertion and the genome. The lower panel shows the PCR products on agarose gel. Primer pair 1 and 2 detect the de novo ZAM insertion at position chrX:10.241.013 and a control primer pair C detects a fixed ZAM insertion (detected in all our libraries) at position chr2L:19.841.922. (**C**) Schema depicting the experimental set up of the single and successive piwi-sKD. (D) Bar plot displaying the TE load for the depicted ERVs in no-KD and after 30, 41 and 72 generations of successive piwi-sKD (G30, G41 and G72). The F2 generation after the last piwi-sKD was analyzed by qPCR. The mean and standard deviation from three biological replicates is shown. The *P*-values where calculated with a two-tailed *t*-test (*P < 0.05, **P < 0.01, ***P < 0.001).

Our analysis so far indicated that despite a general accumulation of ERV transcripts upon piwi-sKD, only few families can proceed in their replication cycle and cross the cell-cell border into the germline. The ultimate proof for transposition activity of an ERV family is to detect new germline insertions. We therefore performed paired-end sequencing of pooled embryos laid by the daughters of piwi-sKD-treated mothers (G1-F2 in Figure 4C). To detect TE insertions, we used the T-lex2 *de novo* pipeline (43,44) that relies on the analysis of unique mapping soft clipped reads and one end anchored (OEA) read pairs from the TE-genome border at the TE insertion site. We then selected insertions specific for either the piwi-sKD or the no-KD condition and calculated the *de novo*-insertion-ratio (piwi-sKD versus no-KD). We obtained a ratio >3-fold for ZAM and gtwin (Figure 4A and Supplementary Figure S3B), two elements showing particularly strong effects of Piwi depletion on piRNA loss, transcript-level increase and eccDNA forms. The naturally occurring transposition rate for TEs is low and ranges between 10^−4^ and 10^−6^ transposition events per copy and generation (54). We thus observed a dramatic increase of the transposition rate in the piwi-sKD to about 10^−1^ for ZAM and gtwin. To get additional examples of *de novo* insertions, we decided to sequence 12 individual adult flies from the F2 generation after piwi-sKD. We used the same pipeline to determine *de novo* insertions in the 12 libraries and found a *de novo* ZAM insertion in an exon of the *Hk* gene on the X chromosome in one of them. The probability of observing a new ZAM insertion among 12 progenies of no-KD parents was estimated only 9.6 × 10^−3^ and 9.6 × 10^−5^, depending on the reported natural transposition rates (54). We also performed genomic PCR to confirm that this insertion was actually present in the genome of the piwi-sKD offspring but not in that of the no-KD parents (Figure 4B), validating our bioinfomatic analysis. To further determine if the increased transposition rates observed above would be stable over many generations of TE derepression, and how the TE load for different elements would develop, we performed the piwi-sKD for 70 successive generations. Retrotransposition is replicative, which means that every new insertion increases the TE load in the genome. To determine the TE load in flies after successive generations of piwi-sKD we performed qPCR experiments with TE- specific primers on genomic DNA. We could clearly observe a strong increase in TE load for ZAM and gtwin over generations in two different piwi-sKD fly sublines that were treated in parallel with successive piwi-sKD (Figure 4D and Supplementary Figure S3B). This further confirms the results we obtained with the DNA-seq experiment after one generation of piwi-sKD. All other tested TEs did not display a change in TE load (Figure 4D and Supplementary Figure S3B). We calculated the transposition rate for ZAM and gtwin with the data obtained from the qPCR experiments and found again an increase for ZAM and gtwin to a frequency of about 10^−2^ to 10^−1^ per copy and generation, which was stable during the entire long-term observation period and comparable to the transposition rate calculated in the DNA-seq experiment.

## DISCUSSION

TEs can replicate by inserting new copies in the genome of the progeny. The Piwi-mediated piRNA pathway is assumed to be one of the major genome defense mechanisms that prevent this potentially harmful replication by downregulating the expression of a large number of TE families at the transcriptional level (55,56). However, no evidence for bursts of replicative transposition upon piRNA pathway impairment has been obtained so far. This is partly due to the fact that piRNA pathway mutants are sterile and therefore do not produce the progeny where transposition can be demonstrated.

Here, we restricted the knockdown of Piwi to the follicle cells of the adult ovary to preserve female fertility for studying the impact of this impairment on transposition. Consistent with the existence of a follicle cell-specific piRNA pathway dedicated to TEs of the ERV subclass (29), our tissue-specific partial Piwi depletion led to a dramatic loss of the piRNAs specifically targeting ERV families (Figure 1A). As a result, almost half of the ERV families displayed a more than two-fold steady state RNA accumulation (Figure 1C). Up to 20% of the ERV families that were transcriptionally activated in the somatic ovarian tissues were eventually detected in the germline as embryonic extrachromosomal circular cDNAs (Figure 3B). The transcriptional derepression of an ERV family is indeed not a good predictor of its ability to complete a full ‘infectious’ replication cycle. For instance, the 10 times increase of gypsy transcription in piwi-sKD ovaries did not even result in a detectable accumulation of ovarian Gag and Env proteins (Supplementary Figure S4). Finally, we found new insertions in the genome progeny for at least two of these ‘infectious’ ERV families, ZAM and gtwin (Figure 4A). Their transposition rate per copy per generation was 2–5 orders of magnitude higher than the natural transposition rates previously reported (54) and it has remained unchanged for at least 70 generations of recurrent somatic Piwi knockdown. Eventually, this has led to a 10-fold increase of the genomic copy number, which probably does not yet represent the maximum attainable (Figure 4D). These observations clearly demonstrate that the Piwi-mediated ERV repression in the ovarian somatic cells contributes to genome homeostasis.

For the ERV family members Tirant and gypsy10 a strong piRNA loss did not result in any significant transcript-level derepression (Figure 1C). Two hypotheses can explain this observation: Either the genome of our fly strain does not contain any active elements from these families to be targeted by piRNAs, or they are controlled by other mechanisms that are redundant with the piRNA pathway. The first hypothesis is consistent with the fact that, in contrast to the female germline, the production of piRNAs in the follicle cells does not require the presence of active TE copies (29,57,58). In this tissue, piRNAs are produced by discrete genomic regions called piRNA clusters that are predominantly composed of an accumulation of TE insertions which occurred during the fly strain's evolutionary history. After the invasion of a genome by a new TE family, the host eventually tames the element if a new copy of this family happens to insert itself into a piRNA cluster. This expands the sequence repertoire available to this peculiar genomic ‘immune system’. The targeting of active elements by piRNAs reduces the replicative transposition rate to levels too low to compensate for the mutation/selection-driven gradual loss of active copies. Eventually, active copies of the family become extinct (59). For some families like gypsy, the average number of active copies may be so low that strains devoid of any active gypsy can be derived from natural populations (60). Thus, it is indeed possible that the Tirant and gypsy10 piRNAs are currently useless but may become strongly beneficial to control the active copies that appear during a re-contact, e.g. after introgression from an active strain or after horizontal transfer from another species. The second hypothesis postulates that TEs are controlled by several transcriptional and/or post-transcriptional mechanisms in a redundant manner. The siRNA pathway is one example of post-transcriptional TE control known to operate mainly in the non-gonadal tissues (21–25). In the follicle cells, an increase of 21nt-long siRNA-like RNA upon Piwi somatic knockdown was observed (31). We have shown here that these small RNAs can effectively reduce the steady state RNA level of a number of ERV families in piwi-depleted follicle cells. Indeed, when such cells were co-depleted of Dicer-2, we observed a siRNA loss correlating with increased accumulation of the cognate transcripts for 10 families (Figure 2D). Almost all of these siRNA-controlled ERVs belong to the subset of families that were already derepressed by the Piwi depletion alone (Figure 2E). Indeed, half of the piRNA pathway-regulated ERV families appear to be jointly regulated by both the piRNA and the siRNA pathway. This dual TE repression layer was revealed recently in Drosophila heads (BioRxiv: https://doi.org/10.1101/267039). This is reminiscent of the situation in the germline, where the piRNA pathway regulates TEs at both the transcriptional (via Piwi) and the post-transcriptional level (via Aub and Ago3). The Piwi depletion would release the sense and antisense TE transcription that is necessary to generate the double-stranded RNA precursors of the siRNAs. According to this hypothesis, the other families whose piRNA loss could not be backed up by siRNAs should be devoid of the putative internal promoters responsible for the production of antisense transcripts (61).

Conversely, 5–8 TE families displaying only a very minor change in piRNA level still present a significant increase of TE transcripts. At least three non-mutually exclusive hypotheses might explain this discrepancy but, unfortunately, none of them could be tested experimentally in a straightforward way. First, we note that the expression of some TEs may be especially sensitive to small quantitative variations of the cognate piRNAs. For example, we previously demonstrated that a small variation of the piRNAs targeting the I-element has a strong consequence on the expression of this TE (62). Second, the piRNA loss may have been underestimated for some ERV families. Indeed, the small RNA-seq experiments were done on whole ovaries, containing germinal tissues with an undisturbed piRNA pathway that might have masked the somatically restricted effects of the Piwi depletion. Sometimes, the putative presence of such buffering piRNAs can be suspected when they originate from the germen-specific dual-strand piRNA clusters because they produce both sense- and antisense-oriented piRNAs (see, for instance, the case of 412 in Figure 2A). However, the antisense germinal piRNAs which originate from uni-strand piRNA source loci, such as the 20A piRNA cluster, cannot be distinguished from the soma-specific piRNAs. Third, the piwi-sKD might have affected TE expression *via* some indirect effect on the host gene expression. To test this hypothesis, we have studied to which extent the host transcriptome was affected in the piwi-sKD ovaries (data not shown). After a tentative GO term analysis (using topGO), we failed to pull an unknown TE-specific transcription factor/pathway out of the 4079 genes that were significantly differentially expressed between piwi-sKD and control: neither a putative activator could be pin-pointed among the 1845 genes that were up-regulated (306 more than 2-fold higher) nor a putative repressor among the 2234 genes that were down-regulated (138 more than 2-fold lower).

It has been previously reported that piRNAs targeting the gypsy or ZAM elements can prevent the production of virus-like particles (VLPs) in follicle cells and thus render them unable to invade the adjacent female germline and insert new proviruses into the genome of the progeny (7–9). To study this phenomenon at a genome-wide level, we took advantage of our Piwi somatic depletion system that allowed us to track the effects of a transient impairment in subsequent generations. For four ERVs families, including ZAM, we were able to detect the accumulation of extrachromosomal circular cDNAs (eccDNAs) in the eggs laid by somatic Piwi-depleted females (Figure 3B). These cDNA molecules likely represent dead-end integration products when the viral cDNA is unable to integrate (51–53). Detection of the eccDNAs in early embryos suggests that most steps of the retroviral replication cycle, including translation, viral particle assembly, germline infection, reverse transcription and entry of the cDNA into the infected nucleus were successful (Figure 3A). Even though such eccDNAs are not necessarily correlated with new insertions, they demonstrate three additional examples of active soma-to-germline transfer. Interestingly, one of them, the 412 element, does not encode the Env transmembrane protein that promotes retroviral infection (63). A case of Env-independent ERV germline infection was already reported for gypsy (8). Our observation suggests that either the 412 element uses the envelope of another ERV family to form infection viral particles or that the element is able to hitchhike the vitellogenic pathway and enter the oocyte by endocytosis as suggested for ZAM (7).

We show here that the transient depletion of Piwi in follicle cells is followed by a dramatic increase of the transposition rates of two ERV families, ZAM and gtwin. The identification of these two families is likely a conservative estimate because the DNA-seq analysis may have missed genomic integrations of other ERV families (see Materials and Methods for bio-informatics analyses). Another reason why our study probably underestimated the repressive role of Piwi on transposition, is because it was only restricted to its expression in somatic ovarian cells. Indeed, Piwi was shown to strongly contribute to TE silencing in the female germline (31,55). Moreover, it has also been known to repress the transposition of the mdg1 ERV family in *Drosophila* testes (64).

An exponential increase of the genomic copy number of both ZAM and gtwin was observed during the 70 successive generations of somatic Piwi depletion, reaching a roughly 10-fold increased genomic load at the 70th generation. This means that the replicative transposition rate has consistently exceeded the efficiency of mechanisms decreasing the copy number (e.g. excision, recombination, counter-selection etc.) during this period of time. Therefore, no alternative control mechanism seems yet to have taken over the maintenance of genome integrity. Future experiments will determine whether the increase of TE load within the genome might affect the fitness of the fly population.

## DATA AVAILABILITY

Data are accessible in the NCBI Gene Expression Omnibus (GEO; https://www.ncbi.nlm.nih.gov/geo/) under the accession number GSE112972.

## Supplementary Material

Supplementary Data
