# Supplementary material for: The somatic piRNA pathway controls germline transposition over generations
Source: Nucleic Acids Res. 2018 Aug 24;46(18):9524–36. doi: 10.1093/nar/gky761 (PMC6182186; doi:10.1093/nar/gky761)
Supplement: Supplementary Data [file gky761_supplemental_files.pdf]

# Supplementary Materials and Methods

## Supplementary Tables

Supplementary Table 1

| **Drosophila Stocks and crosses** |
|---|
| #1 : y w ; **tj-GAL4**/CyO ; (DGGR # 104055) |
| #2 : w ; ; P{$w^{+mC}$=**tubP-GAL80$^{ts}$**}2/TM2 (Bloom. #7017) |
| #3 : y sc v ; ; **sh-piwi** (Bloom. #33724) |
| #4 : w ; tj-**GAL4** ; tubP-**GAL80$^{ts}$ sh-piwi** (stock obtained from a single pair of flies after having recombined together the three transgenes from stocks #1, #2 and #3) |
| #5 : y w ; ; P{$ry^{+t7.2}$=neoFRT}82B P{$w^{+mC}$=tubP-**GAL80**}LL3 (Bloom. #5135) |
| #6 : tj-**GAL4** / + ; **GAL80$^{ts}$ sh-piwi** / **GAL80** (females obtained by crossing stocks #4 and #5) |
| #7 : y sc v ; ; **sh-dcr2** (Bloom. #33656) |
| #8 : y v; ; **sh-w** (Bloom. # 33623) |
| #9 : y ; cn bw sp ; (alias iso-1, D. mel genome sequence strain) |
| #10 : y ; cn bw sp ; **sh-dcr2** (result of the isogenization of stock #7 after 3 successive recombinations with stock #9) |
| #11 : y ; cn bw sp ; **sh-w** (result of the isogenization of stock #8 after 3 successive recombinations with stock #9) |
| #12: tj-**GAL4** / + ; **GAL80$^{ts}$ sh-piwi** / **sh-dcr2** (females obtained by crossing stocks #4 and #10) |
| #13: tj-**GAL4** / + ; **GAL80$^{ts}$ sh-piwi** / **sh-w** (females obtained by crossing stocks #4 and #11) |
| #14 : w lncRNA:flam$^{OR(P)}$ (this so-called **wOR(P)** lab stock is permissive for gypsy but **devoid of active gypsy**) |
| #15 : y v f mal lncRNA:flam$^{1(P)}$ (these permissive females, selected from the **N271** lab stock (1) **express active gypsy**) |

Somatic ovarian RNAi was performed by shifting females #4, #6, #12 and #13 @25°C for 5 days from late pupal to adult stage.

Supplementary Table 2

| | Fw | Rev |
|---|---|---|
| Genomic and RT primer for TEs | | |
| ZAM | CTACGAAATGGCAAGATTAATTCCACTTCC | CCCGTTTCCTTTATGTCGCAGTAGCT |
| gtwin | CGGTCATAATCTTAGGGGGGGAG | GGGCGCCAATTAATGTGTTCCT |
| Gypsy | CTTCACGTTCTGCGAGCGGTCT | CGCTCGAAGGTTACCAGGTAGGTTC |
| 412 | CACCGGTTTGGTCGAAAG | GGACATGCCTGGTATTTTGG |
| HMS-Beagle2 | ATTACAATAAACGGTCGCTTGC | ATGTTTTGAATGCCAGGTGC |
| Stalker | GATGACCAGACAGCAAAACG | TTCCAGTGCTTCTCCCAAC |
| Gypsy8 | TGCAGCTCTAACTCCCACCT | AACGCAGCGGAAAGAAACTA |
| Idefix | TGTCCGTCTGCTCAAAAATTC | GGTCTCGCCCTTCTTTTCTT |
| Circe | CCCTCAACTCAACCTGACGTA | TGATCGGTGTTTTCGATTATGA |
| Rpl32 | CCGCTTCAAGGGACAGTATCTG | ATCTCGCCGCAGTAAACGC |
| Gypsy5 | AATGCAACATCGGAGGATTT | CAGGGTTGGGCAGGTAAGTA |

| | | |
|---|---|---|
| Mdg1 | GCCCAAAAATTTCAGGAACA | TTACGAGTGCACTTGCGATT |
| Tabor | ACGTTGTTCACGACATTAGCCG | GGGTTGGTTCGGATCTGACG |
| Gypsy10 | ATCCCGGTCGCCTTACAATA | GCCCGACTTCAGATCAAGAG |
| Mariner2 | GCCTACTTTTCAAGCGGATG | TCTTCGTTTTGTCGCACTTG |
| Copia | CTTTTAGCCGAGCAAGATGTG | CATAAACGGCGTCCAAATTCTC |
| Blood | GGGAATTCTAAACAGCGACAAC | CCCATTACCACACTCTACTCAC |
| 297 | CCAATACATAAACCCACAACCC | GTTCACTGTCCGATGGTCTTAG |
| eccDNA detection | | |
| ZAM | CAATTCCTCCGTCTATGGC | CATTCCAGTTTTCCGGC |
| Validation of ZAM insertion | | |
| ZAM-HK _1 | TTCGCTCAGACAGAACAACG | TAGGCCTCCATGATCTCCAC |
| ZAM-HK _2 | CCGATCAAAATTCTCCCAAG | CGAGAAGCTCTTCGTCTTGAA |
| ZAM-chr2R-1 | GTTCTGGACCCGCATAACTC | AGCTTTTCCGAGCTTTTTCC |
| Quantification small RNAs | | |
| si-gypsy5 | GCAGTTATGTAACTCCTGTAC | GTCCAGTTTTTTTTTTTTTTTAGGT |
| si-412 | CGCAGTTCTGACTGTTGT | GTTTTTTTTTTTTTTTAGGCCGTA |
| pi-HeT-A | CTGACGAATCGCGCT | TTTTTTTTTTTTTTTCGGGTTCCA |
| pi-42A | AGTATCTACGCATCGATCT | GTCCAGTTTTTTTTTTTTTTTAAGGA |
| pi-I-element[1] | CAGTTATTGTTCCGGAACTC | TCCAGTTTTTTTTTTTTTTTCAGA |
| pi-I-element[2] | GCAGTAATAGATTGGTACCATG | CCAGTTTTTTTTTTTTTTTGCAGT |
| mir-14 | CAGTCAGTCTTTTTCTCTCT | GTCCAGTTTTTTTTTTTTTTTATAGG |
| mir-34 | TGGCAGTGTGGTTAGCT | TCCAGTTTTTTTTTTTTTTTCACAA |

Protein extraction and Western blot analysis.

Ovaries from 20 flies were dissected in cold PBS and then mixed with 2× Laemmli buffer (Sigma-Aldrich) and loaded on a 10% acrylamide gel. Proteins were transferred and used for Western blot analysis with the mouse monoclonal anti-gypsy envelope antibody 8E7 (1/300) (2) or the rabbit polyclonal anti-gypsy gag antibody (1/3000). Generation of the polyclonal antibody was done by synt:em (www.syntem.com). The following peptides corresponding to different parts of gypsy Gag were synthesized: (1) CTYSDKTSLRLLRQGLEMVR, (2) EAPKQKDPKEEYEKTAKAA. Two rabbits were immunized with the two peptides according to the manufacturer's protocol. Mouse anti-α-tubulin antibody (Ab7291; Abcam) was also used.

References

1. Touret,F., Guiguen,F. and Terzian,C. (2014) Wolbachia Influences the Maternal Transmission of the gypsy Endogenous Retrovirus in Drosophila melanogaster. *mBio*, **5**.

2. Song,S.U., Gerasimova,T., Kurkulos,M., Boeke,J.D. and Corces,V.G. (1994) An env-like protein encoded by a Drosophila retroelement: evidence that gypsy is an infectious retrovirus. *Genes Dev.*, **8**, 2046–2057.

Supplementary Figure 1

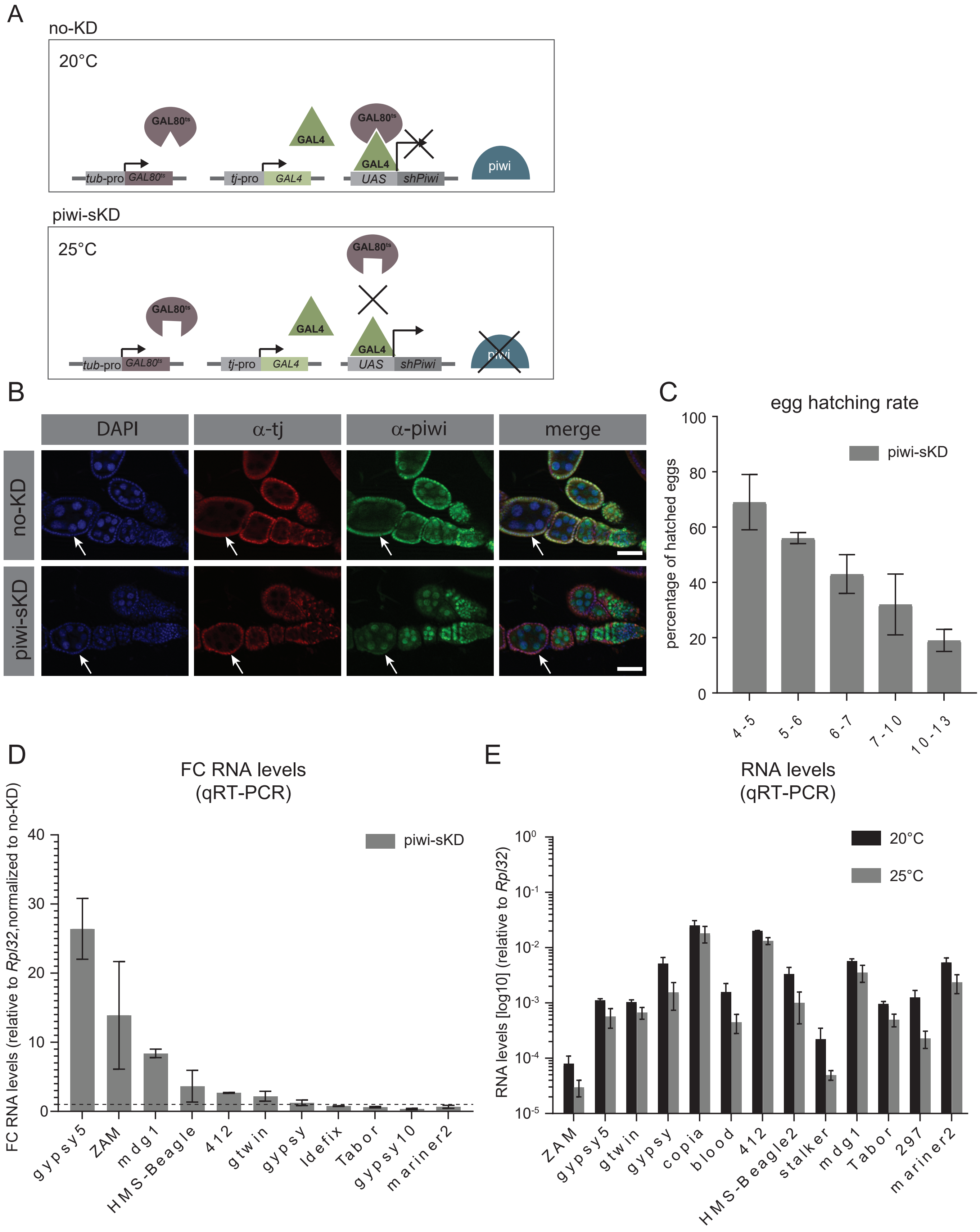

(A) Schematic representation of the no-KD and piwi-sKD condition. $GAL80^{ts}$ (purple ovals) is expressed from the ubiquitous expressing tubulin promoter. At 20°C it sequesters GAL4 (green triangles), expressed from the follicle cell specific tj-promoter, shPiwi is not expressed. At 25°C GAL80 is unable to sequester GAL4, which activates the expression of shPiwi, depleting piwi protein (blue semicircle).

(B) Immunofluorescence of *Drosophila* ovarioles from no-KD and piwi-sKD conditions. Colum 1-3 shows the single channel images for DNA-staining (DAPI, blue), $\alpha$-tj staining (red), to mark the somatic cells and $\alpha$-piwi staining (green). Colum 4 shows the merge of the three channels. The arrow points to the layer of somatic follicle cells surrounding the germ line cells.

(C) Bar plot representing the percentage of hatched eggs laid by piwi-sKD treated females. Piwi-sKD was applied from day 0 till day 13 and the hatching rate measured on egg lays from the indicated time windows, n = 2. Error bars indicate SD.

(D) Bar plot showing relative TE RNA levels for the indicated elements in piwi-sKD ovaries normalized to TE RNA levels from no-KD ovaries set to 1 (dotted line). RNA levels relative to *Rpl32* were obtained by qRT-PCR, n = 3. Error bars indicate SD.

(E) Bar plot showing the relative RNA levels obtained by qRT-PCR for the indicated elements in ovaries from a fly line consisting of the piwi-sKD background with a crossed in temperature stable GAL80 (for genotype see Supplementary Table 1, #6), so the piwi knock down is blocked by GAL80 independently of the temperature. Flies were either raised at 20°C (black bars) or 25°C (grey bars). RNA levels relative to *Rpl32* are shown (log10 scale), n = 3. Error bars indicate SD.

# Supplementary Figure 2

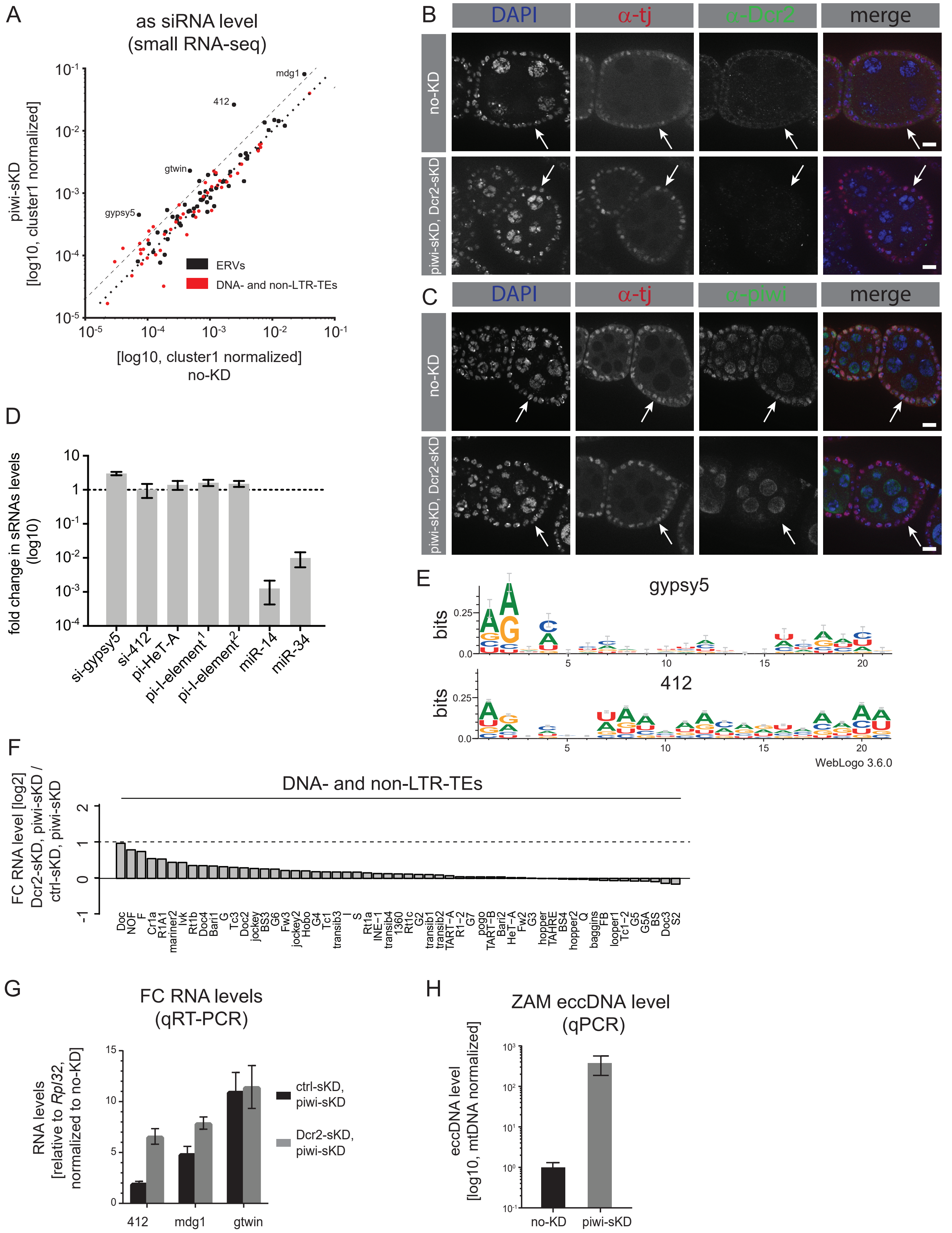

(A) Scatter-plot of antisense 21 nt RNA-seq reads normalized to the number of cluster 1 unique mappers reads (germline specific piRNA cluster) for annotated *Drosophila melanogaster* TEs (n = 123) in piwi somatic knock down (piwi-sKD) versus control (no-KD) (log10 scale; as: antisense). ERVs are depicted as black dotes, DNA- and non-LTR-TEs as red dots.

(B) and (C) Immunofluorescence of *Drosophila* egg chambers from Dcr2- sKD, piwi- sKD and ctrl-sKD, piwi- sKD double knock down conditions. Colum 1-3 show the single channel images for DNA-staining (DAPI, blue), $\alpha$-tj staining (red) to mark the somatic cells and $\alpha$-Dcr2 staining (green) in (B) and DNA-staining (DAPI, blue), $\alpha$-tj (red) and $\alpha$-piwi (green) staining in (C). Colum 4 in (B) and (C) shows the merge of the three. The arrow points to the layer of somatic follicle cells surrounding the germ line cells.

(D) Fold change in the steady-state levels of two siRNAs originating from gypsy5 and 412, three piRNAs derived from HeT-A and I-element (two germline specific transposable elements unaffected by piwi-sKD) and of two microRNAs (miR-14 and miR-34) (see sequences Supplementary Table 2).Total RNA of piwi-sKD ovaries were treated or not by oxidation. Quantification was done relative to a piRNA derived from 42AB (a germline-specific piRNA unaffected by piwi-sKD) (bars represent the mean ± SD of three biological replicates shown (log10 scale).

(E) Weblogo was created by WebLogo 3 version (3.6.0) using 21-nt long reads from piwi-sKD ovaries mapped on gypsy5 (upper panel) and 412 (lower panel).

(F) Bar plot displaying TE RNA level fold changes calculated with normalized read count (two biological replicates) between Dcr2- sKD, piwi- sKD and ctrl- sKD, piwi- sKD double knock down ovaries (log2 scale) for *Drosophila* DNA and non-LTR-TEs (n = 64).

(G) Bar plot showing RNA levels relative to *Rpl32* for the indicated elements in ovaries for Dcr2-sKD, piwi- sKD (grey bars) and ctrl- sKD, piwi- sKD (black bars) double knock down conditions normalized to the respective no-KD condition set to 1, n = 3. Error bars indicate SD.

(H) Bar plot showing eccDNA levels for ZAM relative to mitochondrial DNA (mtDNA) levels in embryos laid by no-KD (black bars) or piwi-sKD (grey bars) mother (log10 scale), n = 3. Error bars indicate SD.

Supplementary Figure 3

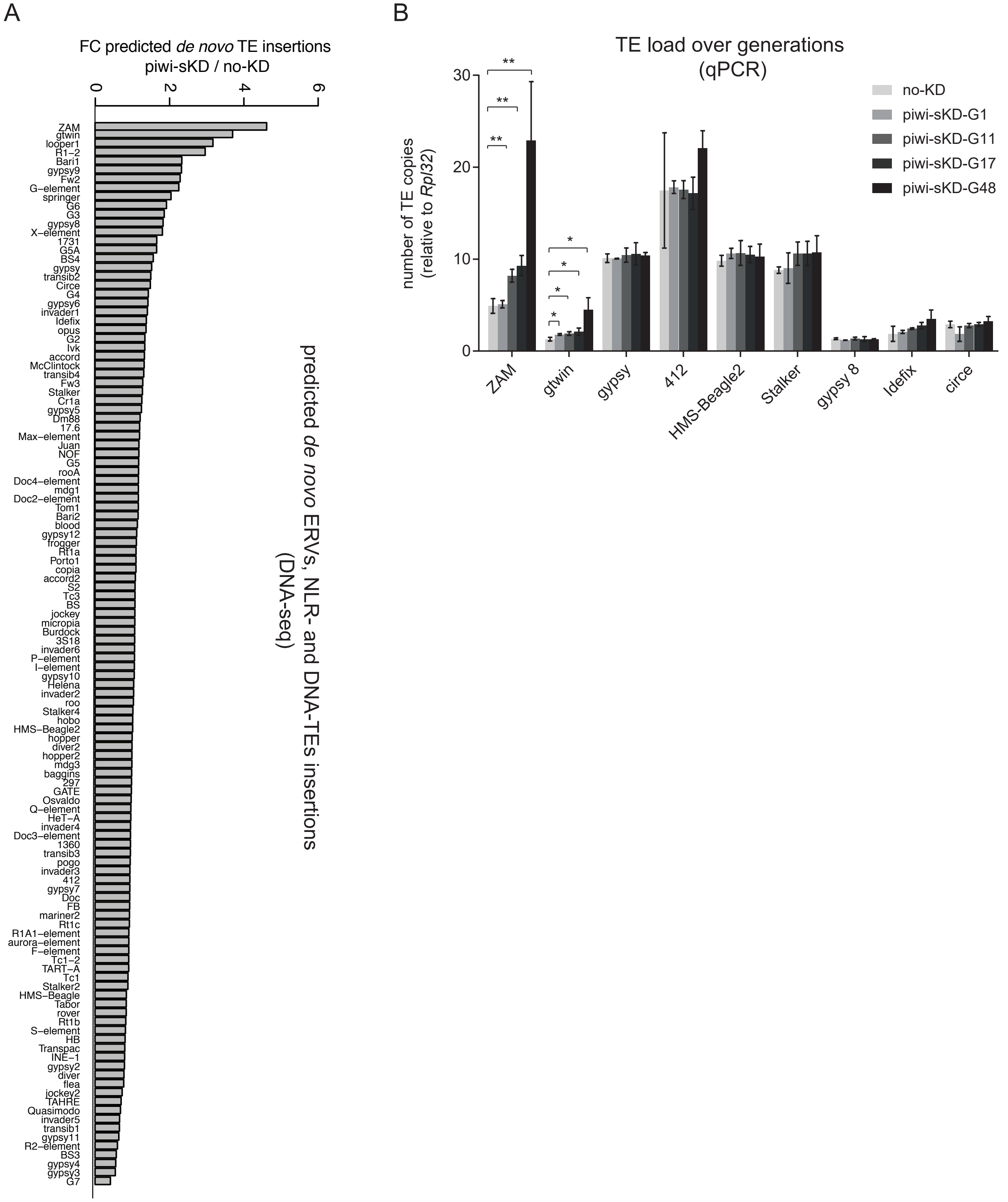


(A) Bar plot displaying the ratio of *de novo* insertions per TE family (n = 123) in embryos of the F2 generation after piwi-sKD versus no-KD embryos.

(B) Bar plot displaying the TE load for the depicted ERVs in no-KD and after 1, 11, 17 and 48 generations of successive piwi-sKD (G1, G11, G17 and G48). The F2 generation after the last piwi-sKD was analyzed by qPCR. The mean and standard deviation from three biological replicates is shown. The p-values where calculated with a two-tailed t-test (* < 0.05, ** < 0.01).

Supplementary Figure 4

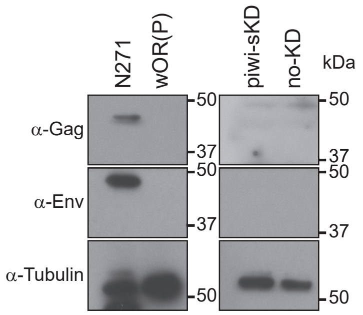


Western blot analysis of proteins extracted from piwi-sKD and no-KD ovaries (right panel). In the left panel, the N271 strain is used as a positive control. These control ovaries contain several active gypsy copies and a permissive flamenco genotype ($flam^P$/ $flam^P$). They originate from a strain which segregates homozygous permissive ($flam^P$/$flam^P$) or restrictive ($flam^P$/FM7 $flam^R$) females for gypsy expression (see Supplementary Table 1). The negative control consists of ovaries from the wOR(P) strain which is devoid of active gypsy. The antibodies used are indicated on the left and the procedure is indicated in supplementary Materials and Methods.
